# Preparing Graduates for Interprofessional Practice in South Africa: The Dissonance Between Learning and Practice

**DOI:** 10.3389/fpubh.2021.594894

**Published:** 2021-02-12

**Authors:** Jana Müller, Ian Couper

**Affiliations:** Department of Global Health, Faculty of Medicine and Health Sciences, Ukwanda Centre for Rural Health, Stellenbosch University, Cape Town, South Africa

**Keywords:** interprofessional, collaborative care, primary health care, hierarchy, clinical training, rural training pathways, health professions education

## Abstract

With South Africa's tumultuous history and resulting burden of disease and disability persisting post-democracy in 1994, a proposed decentralization of heath care with an urgent focus on disease prevention strategies ensued in 2010. Subsequently a nationwide call by students to adapt teaching and learning to an African context spoke to the need for responsive health professions training. Institutions of higher education are therefore encouraged to commit to person-centered comprehensive primary health care (PHC) education which equates to distributed training along the continuum of care. To cope with the complexity of patient care and health care systems, interprofessional education and collaborative practice has been recommended in undergraduate clinical training. Stellenbosch University, South Africa, introduced interprofessional home visits as part of the students' contextual PHC exposure in a rural community in 2012. This interprofessional approach to patient assessment and management in an under-resourced setting challenges students to collaboratively find local solutions to the complex problems identified. This paper reports on an explorative pilot study investigating students' and graduates' perceived value of their interprofessional home visit exposure in preparing them for working in South Africa. Qualitative semi-structured individual and focus group interviews with students and graduates from five different health sciences programmes were conducted. Primary and secondary data sources were analyzed using an inductive approach. Thematic analysis was conducted independently by two researchers and revealed insights into effective patient management requiring an interprofessional team approach. Understanding social determinants of health, other professions' roles, as well as scope and limitations of practice in a resource constrained environment can act as a precursor for collaborative patient care. The continuity of an interprofessional approach to patient care after graduation was perceived to be largely dependent on relationships and professional hierarchy in the workplace. Issues of hierarchy, which are often systemic, affect a sense of professional value, efficacy in patient management and job satisfaction. Limitations to using secondary data for analysis are discussed, noting the need for a larger more comprehensive study. Recommendations for rural training pathways include interprofessional teamwork and health care worker advocacy to facilitate collaborative care in practice.

## Introduction

International studies indicate that effective primary health care (PHC) requires the devolution of hospital-based care into the community, and should include interprofessional collaboration of health professionals to improve patient outcomes (1). In 2010, the South African National Department of Health proposed decentralization of heath care with an urgent focus on disease prevention strategies, early disease detection and intervention at a PHC level in the community (2). Although South Africa is ranked as an upper-middle income country (3), it is the country with the highest income disparity internationally according to the Gini coefficient (4), where poverty, unemployment and related socio-economic factors add significantly to the burden of disease.

The World Health Organization (WHO) defines collaborative health care as a system in which multiple health care workers unite with patients, families, carers and the community to provide comprehensive services of the highest quality across all settings (5). This would imply that health care workers should not only be able to work in interprofessional teams, but also in collaboration with the community they serve. South Africa is considered a melting pot of culture, language, heritage and traditional beliefs, all of which influences how communities function and interact with one another.

Considering the proposed devolution of health care into communities, institutions of higher education had to take conscious steps to adapt curricula in order to train a fit-for-purpose workforce, by introducing PHC training at an undergraduate level (6). Health worker training, which is still predominantly centralized and not distributed along the continuum of care, was further challenged by the nationwide call for relevant and responsive education specific to the African context (7). This call speaks to the need for contextual training based on local clinical problems and health care models. Health care students need to be confronted with the realities, challenges and importance of PHC in a culturally diverse and socio-economic disparate South Africa (8). Finding innovative models for experiential learning that is contextual, transformative and collaborative in nature is an important response to this call. Interprofessional education (IPE), defined as members from two or more professions learning about, from and with each other (9), is a model through which students can develop the necessary skills of working together for improved patient outcomes and also to expose them to the diversity of their peers.

## Background

In 2012, Stellenbosch University (SU) Faculty of Medicine and Health Sciences opened the country's first Rural Clinical School (RCS) campus in Worcester, a town of about 100,000 people situated in the Western Cape Province. This was a timeous response to the need for a national change in the approach to health care service delivery and education (2). The overarching intention of the RCS is to support rural training pathways by providing longitudinal rural exposure and clinical training for undergraduate students focused on social impact and community engagement. This is achieved using hands-on experience of the health issues facing rural, under-served and resource-limited communities in South Africa. The resultant value of this initiative in producing graduates willing to remain in public service and return to rural practice is evident in literature related to the RCS (10, 11). Final year undergraduate Medicine, Occupational Therapy, Human Nutrition, Physiotherapy, and Speech-Language and Hearing Therapy students spend between 6 weeks and 10 months at the RCS, depending on the programme.

In response to a study on the socioeconomic and social capital of a historically disadvantaged and resource-constrained community in Worcester (12), a PHC community-based interprofessional project was piloted from the RCS in 2012. An interprofessional group of academics, health care students from SU, local community organizations and the local community health care workers (CHWs) were involved in the development of the project. The aim of the Collaborative Care Project (CCP) is to expose students to contextually relevant interprofessional learning opportunities that focus on early detection of disease, collaborative management of chronic illnesses and the prevention of disability (13). This is done to aid interprofessional collaborative competency development of students (14), with the aim to foster graduates who work together to promote change. The CCP is an ongoing IPE initiative. Teams of up to five students from different degree programmes based at the RCS and a CHW meet at a health post on a weekly basis to collaboratively plan, conduct home visits and reflect on their experiences. The aim of the interprofessional home visits is for students to collectively identify any previously unreported environmental, personal and health risk factors that patients and their families face. Referrals to appropriate organizations or health care facilities are made by the interprofessional team to provide the household with optimal care in the prevention and management of disease and to address environmental or personal barriers to wellness (15). The collaborative decision-making and writing of referrals promotes engagement during the IPE. The CHWs have intimate knowledge of the community and are expert links between students and families, helping students learn and practice in a culturally sensitive manner. Students reflect at the end of each session in a large group facilitated by a lecturer. They participate in CCP home visits between 6 and 10 times during their clinical rotations in Worcester, which translates to between 18 and 30 hours of interprofessional collaborative engagement.

The available literature exploring students' perceptions of working in interprofessional undergraduate teams in a PHC setting in South Africa largely focuses on competency development and experience of IPE (16). There is a paucity of research in the available literature relating to students' perceptions of practical IPE in preparing them for work in the African context. Such research is necessary to understand better the value of undergraduate IPE in shaping a future workforce (17). This study explored the perceptions of participating undergraduates in the CCP and again as graduates regarding the relevance of IPE in preparing them for work in South Africa. This paper speaks to the factors perceived to impact the carryover of interprofessional collaborative practice into the workplace post-graduation, and the barriers to and facilitators of learning during this project.

## Methods

The first author (JM) was intimately involved in the development of the CCP, and was responsible for coordinating and facilitating the interprofessional patient discussions, home visits and reflections on the process. JM had prior exposure to and knowledge of students' experiences which afforded insight into student perceptions, which was the motivating factor for designing the study. The study adopted a constructivist approach to data collection and an interpretivist paradigm to synthesize and analyze the qualitative data (18).

This study was exploratory in nature and both primary and secondary data sources from individual and focus group interviews were analyzed regarding the relevance and value of the interprofessional home visit project. Primary data were collected through standardized individual telephonic interviews with allied health science (AHS) graduates during 2014 by JM. Secondary data were interview transcripts from two previous research projects investigating students' perceptions of their training at the RCS in 2013, which are described in more detail below.

### Profile of Respondents

Undergraduates who had participated in the CCP during 2013 were interviewed at the end of their academic year and again as graduates during their first year of work in 2014. All respondents were from SU Faculty of Medicine and Health Sciences. They included students from Medicine and the AHS, namely Human Nutrition, Medicine, Occupational Therapy, Physiotherapy, Speech-Language and Hearing Therapy in their final year of study, ranging between the ages of 22 and 35. No undergraduate nursing programme existed at SU during the study period. Of the 76 students who rotated through the CCP at the RCS in 2013, 14 were medical students and 62 were from AHS programmes. See Table 1.

**Table 1:** Numbers of final year undergraduates participating in the CCP during 2013.

**Degree programme**	**Number of students**
Human nutrition	8
Medicine	14
Occupational therapy	8
Physiotherapy	20
Speech-language and hearing therapy	26
Total	76

Most respondents were female, especially from the four AHS professions because of the nature of student intake into these undergraduate professions. The same student cohort was then interviewed as graduates in their first year of working as professionals employed by the national public health care system.

### Sample and Data Collection

For the purposes of this study, primary and secondary data sampling and collection are described separately, as the processes differed.

#### Primary Data

A purposive sample of 20 AHS graduates who had participated in the CCP as undergraduate students were selected by JM to ensure feedback from a representative number of professions. Selection criteria ensured that five graduates were selected per AHS profession with an equal distribution of participants working in rural and urban environments (10 rural and 10 urban). Primary data were collected by individual telephonic semi-structured interviews conducted by JM in October 2014, towards the end of the AHS graduates' first year as working professionals. The interviews were conducted in English and/or Afrikaans and lasted 45–60 minutes. Interviews were recorded, transcribed, translated as necessary and anonymised for analysis.

#### Secondary Data

Secondary data were interview transcripts from two research studies exploring the experiences and perceptions of students training at the RCS.

The first study was a five-year longitudinal study investigating the experience of medical students' training at the RCS, which resulted in a number of publications (10, 11). Semi-structured individual interviews, on average 60 minutes long, were conducted by a research assistant. See the basic interview schedule outlined in Table 2. Data used from this study originated from five individual face-to-face interviews with undergraduate medical students who had spent their entire final year at the RCS in Worcester and two individual telephonic interviews with medical graduates in their first year of practice. The transcribed interviews made specific mention of interprofessional education, the CCP project and/or home visits and were therefore included in the dataset for this study.

**Table 2:** Interview schedules used for primary and secondary data collection.

**Primary data source: semi-structured interviews**
1. Where are you currently working? 2. What kind of work do you do there? 3. How are you involved in interprofessional work? 4. What opportunities do you have to do home visits where you work? 5. What are some of the challenges you face in treating patients holistically? 6. What is communicating and collaborating with other professionals like where you work? 7. How do you feel you contribute to the medical team at the site where you are working? 8. Can you think of a situation where you have applied holistic patient management in your professional career? 9. How do you think the power dynamics in the workplace influence patient's well-being where you work? 10. Tell me about your experience of the interprofessional home visit project as a student? What do you think you've been able to apply in your workplace?
**Secondary data source—Study 1: semi-structured interviews: medical students and interns**
**Medical students:** 1. Tell me about your experiences as an SI at the RCS/longitudinal model? 2. Highlights—special moments 3. Challenges—tough times 4. What about your learning experiences? 5. How did you experience:
Clinical training
Patient-centered approach
Tutorials
Service-learning component
Assessment
6. How did you find working with the hospital staff (might one need to prompt them about other medical staff, nurses, and AHS?) 7. How prepared do you feel for internship? What concerns do you have? 8. What are you hoping to do once you complete your community service? 9. Any other comments?
**Internship placement:** 1. Did you get placed where you wanted to be placed? 2. What has been your overriding experience as an intern since the start of this year? 3. How has the transition from student to intern been? 4. What aspects of your responsibilities did you feel most prepared for? Why? 5. What aspects of your undergraduate training stand out as having been of greatest benefit in terms of your preparedness? 6. What aspects of your responsibilities in the hospital or regarding patient care did you feel least prepared for? 7. What values do you think the MBChB programme taught you? 8. Tell me about the extent to which “external” or community placements affected your preparation for internship, if at all? 9. What have you found most challenging during the past year? 10. Looking back, what do you think could have been different in your undergraduate studies to have ensured that you would have been better equipped as an Intern? 11. To which extent is holistic patient management a feature in the hospital? 12. To which extent did the interprofessional home visits that you conducted last year prepare you for your internship and/or influenced your thinking? 13. What are you hoping to do when you complete your Internship and has this changed over the past few months?
**Secondary data source—Study 2: AHS focus group semi-structured intervie**
1. What are your perceptions (substitute: thoughts) about rural clinical training for your discipline(s)? 2. Reasons for the statements made 3. Examples of perceived experiences 4. People/stakeholders involved 5. Contextual details where the experience(s) occurred 6. Process and strategies that connect to the perception such as teaching, learning and assessment strategies

The second study was an investigation of AHS students' practice at the RCS in 2013 (19). JM was a co-researcher for this study and was involved in all stages of the research project, including data collection and analysis. Data collection included four semi-structured focus group interviews with final year undergraduates who rotated through Worcester during 2013. See Table 2 for the basic interview schedule. Each of the four AHS professions in the faculty (Human Nutrition, Medicine, Occupational Therapy, Physiotherapy, Speech-Language and Hearing Therapy) were represented in this data (See Table 3).

**Table 3:** Distribution of final-year student and graduate interviews analyzed.

	**2013**	**2014**
**Final year student interviews**		
Medical—individual	5	
Physiotherapy—focus group	1	
Occupational therapy—focus group	1	
Speech language and hearing therapy—focus group	1	
Human nutrition—focus group	1	
Total number of final year student interviews	9	
**Graduate individual interviews**		
Medical		2
Physiotherapy		4
Occupational therapy		4
Speech language and hearing therapy		4
Human nutrition		3
Total number of graduate interviews	0	17
Total number of interviews	26	

### Data Analysis

Qualitative data was analyzed using a thematic analysis approach (20) to explore the participants' perceived relevance and value of the CCP. Analysis of all transcripts was conducted by both JM and an independent researcher in rural health professions education, who had no prior knowledge of, or involvement in, the CCP. Each transcript was read, re-read, coded and the emerging data was thematically grouped to systematically identify common threads within the data (20). This was first done individually by the two researchers who then collaboratively agreed upon the final themes. Intercoder reliability was to ensure confirmability of the data by means of investigator triangulation to minimize potential interpretation bias, since JM was also the project coordinator and student learning facilitator for the CCP (21). Written and verbal consent was provided by participants.

Ethical clearance was granted for this study by the Stellenbosch University Faculty of Medicine and Health Sciences Human Research Ethics Committee (HREC) in 2014 (N14/07/094). It was decided not to interview students from the RCS involved in existing studies to avoid over-researching them. Therefore, the data from primary and secondary data sources were utilized; the limitations thereof are discussed later in the paper.

## Findings

A total of 26 interview transcripts were analyzed. This includes the 11 transcripts from the two previously conducted studies and 15 AHS graduate interview transcripts. Due to unreliability of the graduate contact information only 15 of the intended 20 AHS graduates were interviewed.

Themes identified included (1) Appreciation of context (2) Becoming a team (3) Understanding professional limitations and the contribution of other professions (4) Relationships and hierarchy. Table 4 provides a key to the abbreviations used for the respondents.

**Table 4:** Key to abbreviations used for respondents.

**Key to abbreviations:**	
Med	Medical
AHS	Allied health sciences
G	Graduate/professional
S	Student/pre-service

### Appreciation of Context

Student participants reported that involvement in contextual clinical training had an impact on their understanding of the realities of their country, which surpassed the knowledge afforded them by learning in the classroom. “You get to their home, you don't know what to expect, and he's in his wheelchair, but in the rural area, there are potholes everywhere. The RDP houses, they have steps to go into the house and you can't do that with a wheelchair. And that is the community for whom the (state supplied) RDP houses were built. Most of those people in the wheelchairs don't have money to build ramps.” S.AHS4.

The influence social determinants of health have on patient health became real to the students during home visits: “You need to ask (the patients) about their life, how they cope at home. I understand 100% that's why we do home visits. You're shocked to see, oh hectic, wow, this is bad. This is why my patient didn't arrive (for follow up) or this is why they don't have money, or why they want a (social) grant, they really just can't get by.” (translated) S.Med4. This insight resulted in contextual, holistic assessments and relevant patient management. “Seeing this is where they live and this what they have to deal with, and this is what they have available to them. So, I think that rounds you more as a clinician, to see how you can treat a patient within their setting and what would be more appropriate for them.” S.Med3.

“The highlights for me was always the home visit. I think the therapy is much more concentrating on what the people need, or you see first-hand where they struggle, and you can make a difference there. When you see them in a clinic and you ask them what they struggle with you can't always give them a solution they need, a practical solution you either don't understand or can't see or doesn't make sense to you. So practically solving problems with them was definitely the highlight for me.” G.AHS7.

Having clearer insight into a patient's home and community environment helped participants reimagine treatment plans along the continuum of care in a resource constrained environment.

“After being on the home visits you are constantly asking okay, what does the home environment look like, like do they have stairs at home, where is their bathroom. So, I think it has helped you in that regard to think out of the box, not just to think can they get out of the hospital but can they function at home.” G.AHS14.

Affording students the opportunity to interact with their patient's context and culture was an important step in helping achieve the goal of improving competency and confidence as a clinician working in a resource constrained environment. “I think the fact that we are asked to make a difference in someone's life, and you don't feel confident about it. Just going with the mind-set of trying to help people in any way possible and not thinking you gonna save the world, but rather making small changes for people, and I think that is what we miss most of the time as an undergraduate.” G.AHS7. Based on these experiences, participants became more realistic about their expectations of themselves and their patients as well as learning to make use of the resources available to them. “You can't think that you need to get this patient with a CVA (cerebrovascular accident) to walk in a week, which is not realistic in their context. Not going in thinking that there will be equipment and thinking there will be everything that you want and what you think you will need for therapy, but where they can use anything in their homes.” G.AHS7.

“We get to function in the system, and I think that's very valuable, exploring everything that is available instead of just doing robot monotonous kind of work.” S.Med11.

### Becoming a Team

The value of practically working together in the clinical environment was perceived as beneficial in optimizing IPE opportunities and promoting collaborative patient management. “In varsity you don't really get the chance to learn about other disciplines, and when you do it in a work context then it is much easier to see practically what to look at, how they see a patient differently.” G.AHS7

“If you work together, physically work together going on a home visit. You are forced to work together, because while you are an undergraduate you think quite in the box and not out of the box and it really helps to have people with you.” G.AHS4.

Participants reported a cultivation of respect and interest in their own, but also other professions, where they reported feeling comfortable knowing who they should approach in the team for assistance and how to do that. “It is definitely helpful just being more comfortable with approaching other people and referring patients, you know what the other disciplines do, so you are not blindly referring or not referring, because you don't know what a physio or dietician do.” G.Med2

Insight into other professional contributions added value to the notion of what being and becoming a team really means and influenced the degree to which young professionals engaged in interprofessional patient care after graduation. “It's not something completely new, you're not completely unexposed to other people and other disciplines so if you have work with them before it's easy to know what part of the patient they have to look at, or what part you have to ask them about or anything like that.” G.AHS7.

“You sort of have the confidence to go out to the pediatrician and say, ‘No I have been involved with this before, can we try this? I really think it could help us both.’ It gave me more confidence to do the initial hello how are you, can we do this thing.” G.AHS1.

“I think that benefits me a lot. You can definitely see the difference between people that have worked in the team before and the ones that have not... So, you are not just blindly referring or some of the patients don't get referred to because you don't know what the physio does, or a dietician.” G.AHS2.

The team approach used in CCP to identify and manage health and environmental challenges that patients and their families face was seen as a positive catalyst to foster a sense of belonging and value as part of a health care team. “You are with them (AHS students) with a patient they have been rehabbing, but now they maybe found something that's wrong with the patient that needs medical assistance, and then you realize oh my word, so I am actually of use here.” S.Med3.

“… you're not just someone, you're actually a therapist, basically you're a team member.” G.AHS6.

### Understanding Professional Limitations and the Contribution of Other Professions

The very nature of working together and learning about, from and with each other helped provide a more holistic picture of the patient's situation. “Speech got involved and asked random questions that we never heard before. So, we got to see what they did and where they could help our patient. It really helped us with regards to seeing your patient as a whole person.” S.AHS1.

Having insight into the complex nature of a patient's well-being afforded by working as an interprofessional team challenged individualized management approaches. “So, you know up unto where you can help the patient and where you have to draw on other team members to help the patient, because you can't do everything yourself. Where the professions fill each other or complement each other” G.AHS6.

“It shapes better clinicians and teaches us how to work in interdisciplinary teams, which is very important, because the thing is, it would be ideal for South Africa to follow an interdisciplinary model, as a patient would get better treatment.” G.Med1.

Understanding and accepting professional limitations and being part of a cohesive group who were like-minded with shared meaning created a valuable learning environment that contributed to the development of skills required for a sustainable workforce. Graduates reported understanding their own limitations and expressed a sense of relief that there was more that could be done beyond what they knew within their professions. “I mean that's medicine for me (the interprofessional home visit project) and that's where we are going to be when we are out there next year, that's what we are going to see and that's what we will have to be able to do. I mean, you are going to burn yourself out if you think you are going to do everything on your own (chuckles). So, for me I think that's how medicine should be.” S.Med7.

This understanding of professional limitations and contributions of team members included the allocation of roles and responsibilities depending on the patients' needs and not basing leadership on the traditional hierarchical model of doctor as team leader. One medical student put it as follows: “But it was also nice to just have someone else leading for once, because especially when you're in hospital, you see the doctor is usually the one who takes charge and leads the team. But then we have been on home visits where the physio student is the one who is leading, allocating tasks, and asking for everybody's opinion. So, it was nice, it was actually refreshing to see other people also taking the lead.” S.Med3.

The value of the CHWs was recognized, not only as being an integral part of the health care team, but also as co-facilitators of learning, especially with regards to students' exposure to and understanding of the community. “Definite highlights for me were working with the community health care workers. Just getting to know them. … They know the patients on a more intimate level. I felt that they were more accepted in the community, they were more accepted by the family than we really were.” G.AHS7.

Graduates reported that the quality of care they could provide patients as individuals was hindered by the inherent limitations of their scope of practice. This was markedly so with regards to a lack of exposure to dealing collaboratively with social issues in a resource constrained environment. This affected the perceived impact the graduates felt they could have. “The patients come with all their social issues, and I can't really do anything about the social issues, so I get frustrated, and although the social issues have got an influence obviously on their physical illness.” G.Med9.

“(teach us)… how to protect these kids, because some of these kids get abused or raped, but we have never had counseling; I don't know how to counsel a patient. We are not psychologists or counselors, but how to maybe deal about the things that affects our therapy, which is not medical, but is more emotional or social, because that is the hardest for me.” G.AHS12.

The need for a comprehensive understanding of the contributions of other professions and the existing health and social care structures was highlighted by the following quote.

“I think yes, we worked a lot with the PT's, OT's and the doctors, but not much with the social workers, I think we never went into the social side of patient, because over here that hit us really hard and its impact on how you're able to apply your therapy on patients going home and not coming for therapy, either they've been hit by their boyfriends, crossing rivers, doesn't have money, so I would have liked if we could have maybe worked more with social workers or with people would help more on that side, because what is the point of educating a patient on strengthening your right limb when you don't have food and transport to come to therapy... also learning more about the other places you could refer to for these people to get help or assistance which is not medical, talking about care centers, special schools stuff like that besides therapy.” G.AHS6.

One student commented that the experience of working with other professions during the CCP enabled her to consider moving outside her scope of practice and develop specific skills to be better equipped as a therapist in a resource constrained environment.

“I am a Speech Therapist, but there are some other things I can work on, for example balance and muscle strengthening … be more flexible with what you can do and maybe read up about seating or movement. Things that actually may not have anything to do with us, but you have to equip yourself to be more of a general practitioner.” S.AHS2.

Although there was perceived value in learning about what other professions actually do, whether exposure to IPE changed future practice was not always clear. “So that was good to see oh, this is what you are actually doing. Whether that changes my management now I'm not too sure, because I would still refer to the physio or the OT. Yes, maybe I have a better idea now what they are doing, and in that way yes, maybe my thinking is a bit different.” G.Med9.

### Relationships and Hierarchy

The continuation of an interprofessional approach to patient management as graduates was dependent on multiple factors such as relationships, hierarchy and/or a sense of agency.

Relationships among different professionals in the health care team seemed to influence the amount of interprofessional collaboration during the graduates' first year of work and this depended heavily on the responsiveness and availability of doctors and/or senior staff as well as opportunities to connect with one another and be part of a collective. “There was never the opportunity to necessarily bond with them (the doctors) or to visit them or to feel that you can be friends with them.” (translated) G.AHS9.

This was in contrast to another graduate who spoke about “a cool group of doctors that we have, because they even ask for suggestions and what we think and call us and talk about patients over the phone with us.” G.AHS7.

Often graduates felt disempowered by their status as newly qualified professionals to initiate interprofessional collaboration or make recommendations for holistic patient management. This was primarily due to their perceptions of hierarchy and professional status and their comments reveal negative perceptions of their role in the professional team.

“… but you are lower than them, that you're younger and don't have experience.” G.Med4.

Frequent references were made by allied health professionals to “feeling inferior to” or “submissive to” doctors during their first year as working professionals.

“We have wards rounds once a week, but it's more where the doctors speak to the patients mostly and if they feel they want to know something from the physios they ask, but the doctors here are different, we are like the last resource. They're not so keen on the allieds.” G.AHS6.

“… we (allied health therapists) are the bottom of the food chain” (translated) G.AHS8.

“They (medical doctors) are above us, so it didn't even feel as if we (allied health therapists) were halfway equal to them.” (translated) G.AHS1.

Interestingly, there was a negative perception of medical undergraduates' understanding of contributions of the rest of the health care team, which seemed to be a motivating factor for earlier IPE. “Allied Health wants to spend more time with the medical professions from an earlier stage, not because they don't know what medicine does, but medicine doesn't know what they do.” G.AHS2. The value of interprofessional exposure to reduce ignorance relating to other professions also seemed to contribute to the notion of seeing students from other professional programmes as “people.” “I have especially been fascinated by occupational therapists. I didn't realize they can do so many different things. It's only now that you get to see what they do, and also just interacting with them in the hostel as well, getting to know them as people and getting to understand what they go through as well as students.” S.Med4.

Despite AHS graduates' experiences during the CCP working with medical students or leading interprofessional patient management, not all graduates felt comfortable initiating interprofessional communication. I: “And from your experience last year, did it make it any easier for you to talk to the doctors this year?” R: “I wouldn't say any easier. Here at our hospital for example we try to have as much contact with the doctors as well, but they are not always available.” G.AHS9.

There were graduates whose sense of agency enabled their participation in the health care team.

“What we did this year, we just sort of included ourselves in their world (the medical doctors' world), going to morbidity and mortality meetings, doctors' ward rounds, things like that. So we were there. We sort of bring ourselves to the forefront that is the easiest way.” G.AHS1.

Some graduates commented on the need to maintain their internal Monologues to empower themselves in the workplace. “You must be confident in your profession. Although doctors perhaps they have the knowledge of other things … you must still think I am the speech therapist, I am the occupational therapist and I know about this… You must give your opinion to them and not feel submissive to.” (translated) G.AHS4.

In responding to a question regarding the advice they would give to graduates wanting to work in interprofessional teams but struggling with initiating communication, based on their experience, a participant stated:

“I'd probably tell them to invest in getting to know people and being involved as much as possible and forcing your way into ward rounds like I have. I would just pitch up and be like, ‘can I please join your ward rounds?’ and it's hard for them to say no.” G.AHS10.

In order for there to be adequate exposure to other professions and ways of working within the health care system, graduates felt that practice-based IPE happened too late in their training and suggested that the relationship development between student professions be threaded throughout undergraduate training to help foster and establish relationships for life.

“I think not just to implement that multi-disciplinary thing only in your final year, but throughout varsity so that the doctors see what the physios do and the OTs and whatever and work together more in your university years instead of just in the hospital. I think that is something that will help that you see that person as someone who complements your profession, not just someone you can use if you want to. Because I often feel that's the idea they have, they are there, that is fine, but we can also do that.” G.AHS9.

## Discussion

The findings indicate that students' involvement in the collaborative care project (CCP) helped them understand more about their own and other professionals' roles in the team and how a team approach contributes to improved patient care and potentially job satisfaction. The results also indicate that students who understand their role and scope of practice within a team have better discharge planning and continuity of care. These findings are in line with the existing literature on the value of interprofessional collaborative care (22–24). However, the extent to which this knowledge impacts on practice as a graduate appears to be limited (17).

Having a defined scope of practice in a resource constrained environment, where the presence of professionals with other skill sets may be limited, affected the extent to which graduates felt competent in managing the complexity of patient care. The limited contribution they felt able to make to patient-centered care in the absence of an interprofessional team was tangible. These challenges can, in part, be addressed by helping professionals develop diverse skill sets to address their feelings of inadequacy when confronted by the burden of disease and lack of health care resources (1, 25). Professionals with a broader skills set than their own is what Rhoda et al. (26) refer to as a “T-shaped graduate,” where the graduate not only has deep insight into his/her profession, but also basic knowledge and skills of other professions that could assist them in their work with patients and families in South Africa. Alternatively, improved interprofessional relationship development between professions in resource limited settings could enable remote collaborative discussion and idea sharing regarding patient management, as demonstrated by the CCP.

Curricula aimed at helping undergraduates develop higher-order thinking around their role as transformative, adaptive, self-confident health care team members are important (27), especially given the existing traditional hierarchies, siloed patient management and limited resources (28, 29). Graduates to traverse the disparate worlds of state and private health care, urban and rural areas, discrepancies in staff to patient ratios as well as the range of burden of disease for varying communities in South Africa and in many lower-middle income countries (LMICs) (1, 30). Interprofessional collaboration in a complex, disparate health care system is non-negotiable. However, considering that only a small percentage of SU's undergraduates experience practical IPE via the CCP, it is unrealistic to expect graduates to relinquish or share control of patient care in a team-based approach. Exploring what exposure a fit-for-purpose health care graduate needs to navigate any given context as empowered change agents is important for improved patient outcomes.

Despite the exposure and evidence of learning described by the participants, it is clear that the carryover of interprofessional practice into the workplace is not always possible and depends on relationships, hierarchy and agency, which mirrors the findings from international literature (17). Role modeling and professional hierarchy at sites where graduates work have been shown to have an important influence on agency in the workplace and interprofessional collaboration (17, 31, 32). It appears that the lack of awareness of other professions' contribution to patient management may add to the traditional models of hierarchy in the workplace after graduation. Literature indicates that even in clinical environments where physicians believed the departments they worked in were not hierarchical, issues of hierarchy are still evident (33). The very nature, understanding and enactment of collaborative practice is a challenge if hierarchy persists (33, 34).

Role models are not always available in the clinical setting and some graduates felt disempowered to act as change agents and encourage interprofessional collaboration in their working environments. The graduates' sense of agency seemed to influence the extent to which they would use and expand on the existing interprofessional engagements at the hospital, which is also evident in other contexts (17). The notion of being someone “you can just use,” of being “lower than” or not “powerful enough to initiate changes” within a system or even to make recommendations regarding patient management is a concern. Perceptions such as these resulted in graduates feeling afraid to appeal to senior staff for assistance when needed, and may hamper skills development, patient care and establishing future professional relationships. Research shows that these factors influence the prevalence of burn-out and disillusionment within the public health sector (25, 35, 36). They have also been shown to have a direct influence on patient experiences and continuity of care (25, 32). Students and graduates who are exposed to institutional hierarchy may construct marginalized identities and perpetuate patterns of power and hierarchy in the workplace. Patient management is often dominated by a specific person, most commonly a doctor, as a result of the socialization of medical and health science students in organized tertiary education settings (37). Alternatively, patient care can take the form of collaboration allowing for interdependent participation and decision-making. This is dependent on the exercise of collaborative power by individuals in the group (37) and should be a consideration when planning rural training pathways.

Understanding how students interpret hierarchy is important, especially since the role they assume as part of the team is often context specific (17). Exposing students to the complexity of PHC so that they understand the value of an interprofessional approach in improved patient care and job satisfaction is one thing, but enabling students to graduate as transformative change agents in a hierarchical system is quite another. Health professions education has to speak to workforce sustainability by adopting approaches that empower graduates to initiate and engage in collaborative patient care in resource constrained environments.

Having a clear understanding of the intended purpose of IPE is necessary to determine the extent to which one can expect graduates to engage in collaborative care or initiate change within existing health care systems. The discourse of collaborative care has been described as being either utilitarian (about achieving better patient care) or emancipatory (about working better together and disrupting hierarchy in systems to improve outcomes) (38). In reflecting on the CCP, the primary aim of the project is utilitarian, and it does not, as yet, actively aim to address issues of hierarchy or competencies that are emancipatory in nature.

Considering this, undergraduate training will need to strive at capacitating students and graduates to navigate systemic hierarchy and power relations in order to build interprofessional relationships if there is to be a change in existing dynamics (39). It should be noted, however, that institutional structures affect the extent to which non-hierarchical collaborative practice is possible as a team, given that established traditions and systems are not easily changed (37, 40).

The results of this study echo previous research that emphasize the need to implement practical IPE earlier in undergraduate training and that it should be an integral part of patient care and clinical education and not seen as an add on, nor should it be considered simply part of the “hidden curriculum” (41–43). The emphasis on improving collaborative practice should focus not on students alone, but also on clinicians and institutions. There is a need to reflect on the institutionalized values and beliefs that have been adopted as the norm if we are to disrupt the traditional hierarchy that exists in the health care system (17, 37, 44). This can only be achieved through relationships developed in co-location whereby mature interprofessional collaboration is the norm and practiced by both academics and clinician role models (39). Teaching students and their preceptors to adopt a collaborative inquiry approach to patient management is a possible strategy which has shown to be effective in various professional teams from the medical to the aviation industry (34). This approach to collaborative care allows for a cycle of decision making, reflection, group inquiry, reasoning and planning, where members of the team adopt a self-correcting dialogue as part of their advocacy and patient care (34, 45).

### Limitations

Although this study is limited to the experiences of a select group of students in a specific context, it does echo the results found in previous international literature. The experience of graduates has limited transferability, not only due to the small number of participants, but also to the challenges of using secondary data for analysis where more pointed questions relating to IPE could have been asked. There is a paucity of literature available on the transfer of IPE to collaborative practice after graduation and a more robust and rigorous study of the CCP following this pilot study could contribute to that literature (17). It is therefore recommended that a larger study with purposively selected, representative samples interviewed with the specific intention to explore the perception of the CCP be done, both at an undergraduate and graduate level. Assessment of interprofessional competency development during IPE is also recommended.

JM's relationship to the project as one of the original facilitators and the coordinator of the CCP since 2012 would have influenced the interpretation of the data and contributed to the depth of understanding and construction of the findings. This was dealt with through reflexivity and by involving an independent researcher from a different ethnic, socio-economic and cultural background to analyze and critically compare findings to those of JM. IC, who acted in a supervisory capacity during the analysis of the results in relation to the existing literature, was not involved in CCP at the time of the research and played a crucial role in ensuring critical reflection during the writing of this article. The primary and secondary data being collected by different interviewers, with their own interview schedules and prompts would have influenced the richness of data regarding the CCP, though the fact that the project was raised spontaneously as a significant aspect of learning has significant. Thus, the results may not be representative of the entire student or graduate group. Using an explicit theoretical basis and exploring the influence working environments have on graduates' perceptions would give a richer understanding of the perceived value of the project (17, 46).

The insight gained from this study could inform further research into understanding professional development in context, how to best prepare students for working within the existing health care system and the value this affords professionals working in resource constrained environments. Understanding how to reinforce a professional's sense of belonging and relationship despite power and hierarchy in the workplace is crucial in terms of the sustainability of collaborative practice and needs further exploration.

## Conclusion

All universities have a responsibility to educate students in relevant, representative contexts to equip them to manage their patients collaboratively (47). Exposure to a variety of communities and cultures is necessary for students to develop insight into the health care needs of populations and be able to adapt their management plans accordingly. The study findings suggest that undergraduate IPE, which is clinical and contextually relevant, has merit in improving comprehensive patient evaluation, management and discharge planning, but it needs to happen earlier in students' training. Graduates who understand the value of, and know how to work in an interprofessional team are more likely to manage patients holistically, feel part of a system, have more confidence and feel less overwhelmed by the complexity of patient care in under resourced settings. Exploring the influences on graduates actioning interprofessional collaborative care in resource constrained environments would be valuable future research. The findings of this study reflect the limitations that health care environments place on the actual practice of collaborative patient care and the extent to which this is possible in health systems where hierarchy and siloed patient management are the norm. If patients with complex challenges are going to be managed effectively in low resourced PHC settings, a critical reflection on the training and experiences students and graduates are offered is necessary.

## Data Availability Statement

The raw data supporting the conclusions of this article will be made available by the authors, within the limitations of the ethical clearance granted.

## Ethics Statement

The studies involving human participants were reviewed and approved by Stellenbosch University Faculty of Medicine and Health Sciences Human Research Ethics committee. The patients/participants provided their written informed consent to participate in this study.

## Author Contributions

JM has been one of the original project facilitators since 2012 and is the coordinator of the Collaborative Care Project (CCP). She was responsible for the planning of the research study and was involved with data collection and analysis. IC acted in a supervisory capacity, was not involved in the CCP and played a crucial role in ensuring critical reflection during the writing of this article. All authors were involved in the writing of this article.

## Conflict of Interest

The authors declare that the research was conducted in the absence of any commercial or financial relationships that could be construed as a potential conflict of interest. The handling editor is currently organizing a Research Topic with one of the authors IC.
